# Intrinsic dynamics induce global symmetry in network controllability

**DOI:** 10.1038/srep08422

**Published:** 2015-02-12

**Authors:** Chen Zhao, Wen-Xu Wang, Yang-Yu Liu, Jean-Jacques Slotine

**Affiliations:** 1 School of Systems Science, Beijing Normal University, Beijing, 10085, P. R. China; 2 Channing Division of Network Medicine, Brigham and Women's Hospital, Harvard Medical School, Boston, Massachusetts 02115, USA; 3 Center for Complex Network Research and Department of Physics, Northeastern University, Boston, Massachusetts 02115, USA; 4 Nonlinear Systems Laboratory, Massachusetts Institute of Technology, Cambridge, Massachusetts, 02139, USA; 5 Department of Mechanical Engineering and Department of Brain and Cognitive Sciences, Massachusetts Institute of Technology, Cambridge, Massachusetts, 02139, USA

## Abstract

Controlling complex networked systems to desired states is a key research goal in contemporary science. Despite recent advances in studying the impact of network topology on controllability, a comprehensive understanding of the synergistic effect of network topology and individual dynamics on controllability is still lacking. Here we offer a theoretical study with particular interest in the diversity of dynamic units characterized by different types of individual dynamics. Interestingly, we find a global symmetry accounting for the invariance of controllability with respect to exchanging the densities of any two different types of dynamic units, irrespective of the network topology. The highest controllability arises at the global symmetry point, at which different types of dynamic units are of the same density. The lowest controllability occurs when all self-loops are either completely absent or present with identical weights. These findings further improve our understanding of network controllability and have implications for devising the optimal control of complex networked systems in a wide range of fields.

Complex networks, such as Internet, WWW, power-grid, cellular and ecological networks, have been at the forefront of complex system studies for more than a decade^1^,^2^. Universal principles that govern the topology and evolution of complex networks have significantly enriched our understanding of them^3^,^4^. Fairly recently, controlling complex networks to desired final states has been a very hot research topic in complex system studies^5^,^6^,^7^,^8^.

As a key notion in control theory, controllability denotes our ability to drive a dynamical system from any initial state to any desired final state in finite time^9^,^10^. For the canonical linear time-invariant (LTI) system 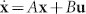 with state vector 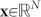, state matrix 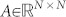 and control matrix 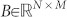, Kalman's rank condition (i.e., 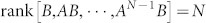) is sufficient and necessary to assure controllability. Yet, in many cases system parameters are not exactly known, rendering classical controllability tests impossible. By assuming that system parameters are either fixed zeros or freely independent, structural control theory (SCT) helps us overcome this difficulty for LTI systems^11^,^12^,^13^,^14^,^15^. Quite recently, many research activities have been devoted to study the structural controllability of linear systems with complex network structure, where system parameters (e.g., the elements in *A*, representing link weights or interaction strengths between nodes) are typically not precisely known, only the zero-nonzero pattern of *A* is known^5^,^6^,^16^,^17^,^18^,^19^,^20^,^21^. Network controllability problem can be typically posed as a combinatorial optimization problem, i.e., identify a minimum set of driver nodes, with size denoted by *N*_D_, whose control is sufficient to fully control the system's dynamics^5^. While the intrinsic individual dynamics can be incorporated in the network model, it would be more natural and fruitful to consider their effects separately. Hence, most of the previous studies focused on the impact of network topology, rather than the individual dynamics of nodes, on network controllability^5^,^17^. Other control related issues, e.g., energy cost of control, have also been extensively studied for complex networked systems^22^,^23^,^24^,^25^.

If one explores the impact of individual dynamics on network controllability in the SCT framework, a specious result would be obtained—a single control input can make an arbitrarily large linear system controllable. Although this result as a special case of the minimum inputs theorem has been proved^5^ and its implication was further emphasized in Ref. 26, this result is inconsistent with empirical situations, implying that the SCT is inapplicable in studying network controllability, if individual dynamics of nodes are imperative to be incorporated to capture the collective dynamic behavior of a networked system. To overcome this difficulty, and more importantly, to understand the impact of individual dynamics on network controllability, we revisit the key assumption of SCT, i.e., the independency of system parameters. We anticipate that major new insights can be obtained by relaxing this assumption, e.g., considering the natural diversity and similarity of individual dynamics. This also offers a more realistic characterization of many real-world networked systems where not all the system parameters are completely independent.

To solve the network controllability problem with dependent system parameters, we rely on the recently developed exact controllability theory (ECT)^27^. ECT enables us to systematically explore the role of individual dynamics in controlling linear systems with arbitrary network topology. In particular, we consider prototypical linear forms of individual dynamics (from first-order to high-orders) that can be incorporated within the network representation of the whole system in a unified matrix form. This paradigm leads to the discovery of a striking symmetry in network controllability: if we exchange the fractions of any two types of dynamic units, the system's controllability (quantified by *N*_D_) remains the same. This exchange-invariant property gives rise to a global symmetry point, at which the highest controllability (i.e., lowest number of driver nodes) emerges. This symmetry-induced optimal controllability holds for any network topology and various categories of individual dynamics. We substantiate these findings numerically in a variety of network models.

## Results

### Controllability measurement

ECT^27^ claims that for arbitrary network topology and link weights characterized by the state matrix *A* in the LTI system 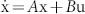, the minimum number of driver nodes *N*_D_ required to be controlled by imposing independent signals to fully control the system is given by the maximum geometric multiplicity max*_i_*{*μ*(*λ_i_*)} of *A*'s eigenvalues {*λ_i_*}^28^,^29^,^30^,^31^,^32^. Here *μ*(*λ_i_*) ≡ *N* − rank(*λ_i_I_N_* − *A*) is the geometric multiplicity of the eigenvalue *λ_i_* and *I_N_* is the identity matrix. Calculating all the eigenvalues of *A* and subsequently counting their geometric multiplicities are generally applicable but computationally prohibitive for large networks. If *A* is symmetric, e.g., in undirected networks, *N*_D_ is simply given by the maximum algebraic multiplicity max*_i_*{*δ*(*λ_i_*)}, where *δ*(*λ_i_*) denotes the degeneracy of eigenvalue *λ_i_*. Calculating *N*_D_ in the case of symmetric *A* is more computationally affordable than in the asymmetric case. Note that for structured systems where the elements in *A* are either fixed zeros or free independent parameters, ECT offers the same results as that of SCT^27^.

### Controllability associated with first-order individual dynamics

**Figure 1:**
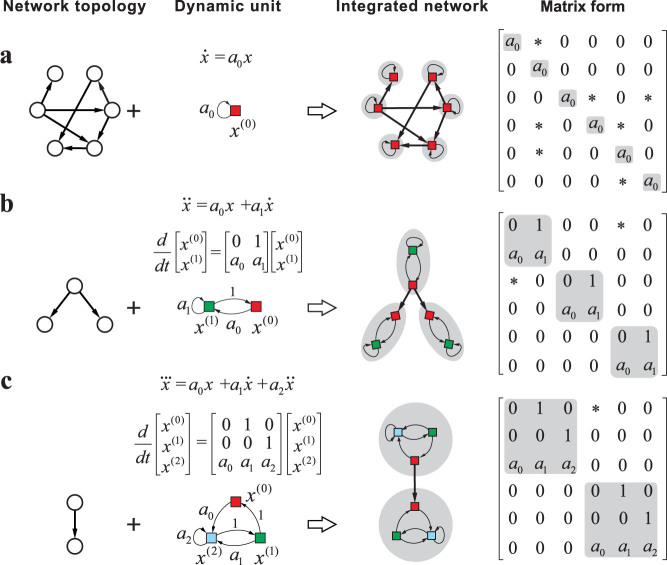
Integration of network topology and intrinsic individual dynamics. 1st-order (a), 2nd-order (b) and 3rd-order (c) individual dynamics. For a *d*th-order individual dynamics 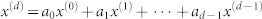, we denote each order by a colored square and the couplings among orders are characterized by links or self-loops. This graphical representation allows individual dynamics to be integrated with their coupling network topology, giving rise to a unified matrix that reflects the dynamics of the whole system. In particular, each dynamic unit in the unified matrix corresponds to a diagonal block and the nonzero elements (denoted by *) apart from the blocks stand for the couplings among different dynamic units. Therefore, the original network consisting of *N* nodes with order *d* is represented in a *dN* × *dN* matrix.

We first study the simplest case of first-order individual dynamics 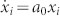. The dynamical equations of an LTI control system associated with first-order individual dynamics^33^ can be written as
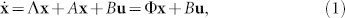
where the vector 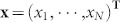 captures the states of *N* nodes, 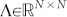 is a diagonal matrix representing intrinsic individual dynamics of each node, 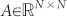 denotes the coupling matrix or the weighted wiring diagram of the networked system, in which *a_ij_* represents the weight of a directed link from node *j* to *i* (for undirected networks, *a_ij_* = *a_ji_*). 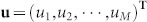 is the input vector of *M* independent signals, 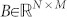 is the control matrix, and Φ ≡ Λ + *A* is the state matrix. Without loss of generality, we assume Λ is a “constant” matrix over the field 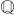 (rational numbers), and *A* is a structured matrix over the field 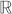 (real numbers). In other words, we assume all the entries in Φ have been rescaled by the individual dynamics parameters. The resulting state matrix Φ is usually called a *mixed matrix* with respect to (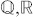)^34^. The first-order individual dynamics in Φ is captured by self-loops in the network representation of Φ (see Fig. 1a). *N*_D_ can then be determined by calculating the maximum geometric multiplicity max*_i_*{*μ*(*λ_i_*)} of Φ's eigenvalues.

**Figure 2:**
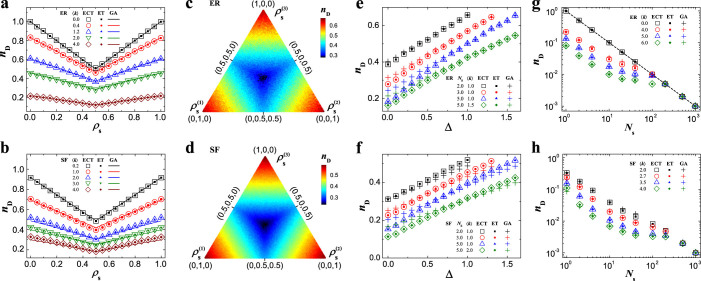
Controllability of networks with 1st-order individual dynamics. (a–b), controllability measure *n*_D_ in the presence of a single type of nonzero self-loops with fraction *ρ*_s_ for random (ER) networks (a) and scale-free (SF) networks (b) with different average degree 〈*k*〉. (c–d), *n*_D_ of ER (c) and SF networks (d) with three types of self-loops *s*_1_, *s*_2_ and *s*_3_ with density 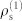, 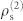 and 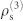, respectively. The color bar denotes the value of *n*_D_ and the coordinates in the triangle stands for 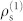 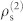 and 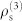. (e–f), *n*_D_ as a function of the density heterogeneity of self-loops (Δ) for ER (e) and SF (f) networks. (g–h), *n*_D_ as a function of the number of different types of self-loops for ER (g) and SF (h) networks. ECT denotes the results obtained from the exact controllability theory, ET denotes the results obtained from the efficient tool and GA denotes the results obtained from the graphical approach. The dotted line in (g) is *n*_D_ = 1/*N*_s_. The networks are described by structured matrix *A* and their sizes in (a)–(d) are 2000 and that in (e)–(h) are 1000. The results from ECT and ET are averaged over 30 different realizations, and those from GA are over 200 realizations.

We study two canonical network models (Erdös-Rényi and Scale-free) with random edge weights and a *ρ_s_* fraction of nodes associated with identical individual dynamics (i.e., self-loops of identical weights). As shown in Fig. 2a, b, the fraction of driver nodes *n*_D_ ≡ *N*_D_/*N* is symmetric about *ρ*_s_ = 0.5, regardless of the network topology. (Note that SCT predicts that in case of independent self-loop weights, *n*_D_ monotonically decreases as *ρ*_s_ augments and eventually *ρ*_s_ = 1 leads to *n*_D_ = 1/*N*, implying that a single driver node can fully control the whole network^26^.) The symmetry can be theoretically predicted (see Methods). An immediate but counterintuitive consequence from the symmetry is that *n*_D_ in the absence of self-loops is exactly the same as the case that each node has a self-loop with identical weight. This is a direct consequence of Kalman's rank condition for controllability^9^:
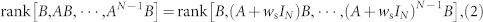
where the left and the right hand sides are the rank of controllability matrix in the absence and full of identical self-loops, respectively (see Supplementary Sec.1 for proof).

The presence of two types of nonzero self-loops *s*_2_ and *s*_3_ leads to even richer behavior of controllability. If the three types of self-loops (including self-loops of zero weights) are randomly distributed at nodes, the impact of their fractions on *n*_D_ can be visualized by mapping the three fractions into a 2D triangle, as shown in Fig. 2e. We see that *n*_D_ exhibits symmetry in the triangle and the minimum *n*_D_ occurs at the center that represents identical fractions of the three different self-loop types. The symmetry-induced highest controllability can be generalized to arbitrary number of self-loops. Assume there exist *n* types of self-loops 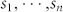 with weights 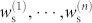, respectively, we have
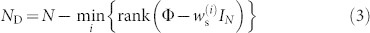
for sparse networks with random weights (see Supplementary Sec.2 for detailed derivation and the formula of dense networks). An immediate prediction of Eq. (3) is that *N*_D_ is primarily determined by the self-loop with the highest density, simplifying Eq. (3) to be 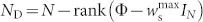, where 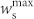 is the weight of the prevailing self-loop (see Supplementary Sec.2). Using Eq. (3) and the fact that Φ is a mixed matrix, we can predict that *N*_D_ remains unchanged if we exchange the densities of any two types of self-loops (see Methods), accounting for the symmetry of *N*_D_ for arbitrary types of self-loops. Due to the dominance of *N*_D_ by the self-loop with the highest density and the exchange-invariance of *N*_D_, the highest controllability with the lowest value of *N*_D_ emerges when distinct self-loops are of the same density.

To validate the symmetry-induced highest controllability predicted by our theory, we quantify the density heterogeneity of self-loops as follows:
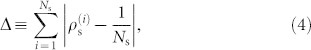
where *N*_s_ is the number of different types of self-loops (or the diversity of self-loops). Note that Δ = 0 if and only if all different types of self-loops have the same density, i.e., 
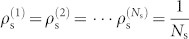
, and the larger value of Δ corresponds to more diverse case. Figure 2e, f shows that *n*_D_ monotonically increases with Δ and the highest controllability (lowest *n*_D_) arises at Δ = 0, in exact agreement with our theoretical prediction. The effect of the heterogeneity of nodal dynamics on the controllability resembles that of the structural heterogeneity discovered in Ref. 5, i.e., more degree heterogeneity leads to larger *n*_D_ and hence worse controllability. Figure 2g, h display *n*_D_ as a function of *N*_s_. We see that *n*_D_ decreases as *N*_s_ increases, suggesting that the diversity of individual dynamics facilitates the control of a networked system. When *N*_s_ = *N* (i.e., all the self-loops are independent), *n*_D_ = 1/*N*, which is also consistent with the prediction of structural control theory^5^.

### Controllability for high-order individual dynamics

In some real networked systems, dynamic units are captured by high-order individual dynamics, prompting us to check if the symmetry-induced highest controllability still holds for higher-order individual dynamics. The graph representation of dynamic units with 2nd-order dynamics is illustrated in Fig. 1b. In this case, the eigenvalues of the dynamic unit's state matrix 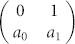 plays a dominant role in determining *N*_D_. For two different units as distinguished by distinct (*a*_0_ *a*_1_) one can show that their state matrices almost always have different eigenvalues, except for some pathological cases of zero measure that occur when the parameters satisfy certain accidental constraints. The eigenvalues of dynamic unit's state matrix take over the roles of self-loops in the 1st-order dynamics, accounting for the following formulas for sparse networks
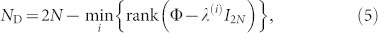
where *λ*^(*i*)^ is either one of the two eigenvalues of type-*i* dynamic unit's state matrix. The formula implies that *N*_D_ is exclusively determined by the prevailing dynamic unit, (see Supplementary Section 2). The symmetry of *N*_D_, i.e., exchanging the densities of any types of dynamic units does not alter *N*_D_ (see Methods), and the emergence of highest controllability at the global symmetry point can be similarly proved as we did in the case of 1st-order individual dynamics.

**Figure 3:**
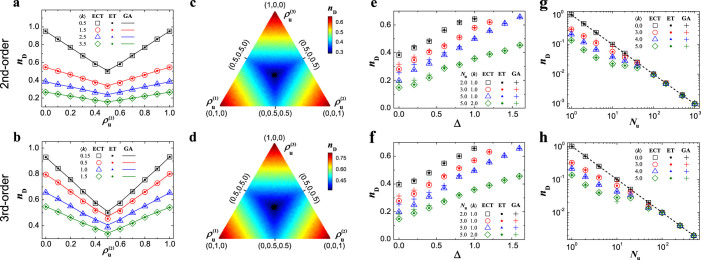
The controllability of networks with high-order individual dynamics. (a–b), controllability measure *n*_D_ in the presence of two types of dynamic units with density 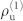 and 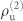 belonging to the 2nd-order dynamic units (a) and 3rd-order dynamic units (b) for ER random networks with different average degree 〈*k*〉. (c–d), *n*_D_ in the presence of three types of dynamic units with density 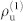, 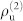 and 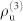 belonging to the 2nd-order dynamic units (c) and 3rd-order dynamic units (d) for ER random networks. The triangle has the same meaning as that in Fig. 2. (e–f), *n*_D_ as a function of the density heterogeneity (Δ) for 2nd-order (e) and 3rd-order (f) dynamic units on ER random networks. (g–h), *n*_D_ as a function of the number *N*_u_ of different dynamic units subject to 2nd-order (g) and 3rd-order (h). The dotted line in (g) and (h) is *n*_D_ = 1/*N*_u_. The network size of the 2nd-order dynamic units is 1000 and that of the 3rd-order dynamic units is 500. The networks are described by structured matrices *A*. The results from ECT and ET are averaged over 30 different realizations, and those from GA are over 200 realizations.

The 3rd-order individual dynamics are graphically characterized by a dynamic unit composed of three nodes (Fig. 1c), leading to a 3*N* × 3*N* state matrix (Fig. 1c). We can generalize Eq. (5) to arbitrary order of individual dynamics:
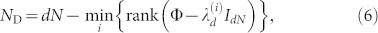
where *d* is the order of the dynamic unit, 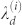 is any one of the *d* eigenvalues of type-*i* dynamic units and *I_dN_* is the identity matrix of dimension *dN*. In analogy with the simplified formula for the 1st-order dynamics, insofar as a type of individual dynamics prevails in the system, Eq. (6) is reduced to 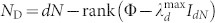, where 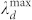 is one of the eigenvalues of the prevailing dynamic unit's state matrix. The global symmetry of controllability and the highest controllability occurs at the global symmetry point can be proved for individual dynamics of any order and arbitrary network topology. Fig. 3 displays the results for 2nd- and 3rd-order individual dynamics, where the density heterogeneity for high-order dynamic units is defined as 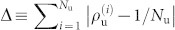, *N*_u_ is the number of different dynamic units and 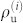 is the density of type-*i* dynamic unit.

**Figure 4:**
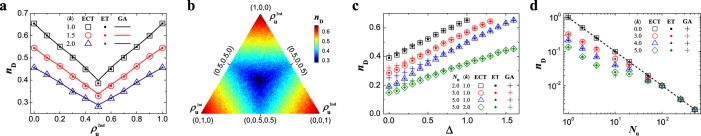
The controllability of networks consisting of a mixture of dynamic units with different orders. (a), *n*_D_ as a function of the density 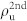 of the 2nd-order dynamic unit incorporated with the 1st-order dynamic units. (b), *n*_D_ as a function of the densities 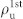, 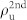 and 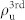 of dynamic units associated with different orders. (c), *n*_D_ as a function of the density heterogeneity, Δ, for a mixture of dynamic units from 1st- to 3rd-order. (d), *n*_D_ as a function of the number *N*_u_ of a mixture of different dynamic units from 1st- to 3rd-order. The number of dynamic units in (a)–(d) is 500 and ER random networks described by structural matrices are used. In (b), the average degree 〈*k*〉 = 1. The dotted line in (d) is *n*_D_ = 1/*N*_u_. Each data point is obtained by averaging over 100 independent realizations. (See Supplementary Section 2 and 3 for ET and GA.)

We have also explored the mixture of individual dynamics with different orders, finding the symmetry of *n*_D_ and the highest controllability at the global symmetry point, in agreement with those found in the networks with single-order individual dynamics (see Fig. 4).

## Discussion

In summary, we map individual dynamics into dynamic units that can be integrated into the matrix representation of a networked system, offering a general paradigm to explore the joint effect of individual dynamics and network topology on the system's controllability. The paradigm leads to a striking discovery: the universal symmetry of controllability as reflected by the invariance of controllability with respect to exchanging the fractions of any two different types of individual dynamics, and the emergence of highest controllability at the global symmetry point. The global symmetry indicates that the controllability is determined exclusively by the densities of different individual dynamics rather than their specific intrinsic dynamics. These findings generally hold for arbitrary networks and individual dynamics of any order. The symmetry-induced highest controllability has immediate implications for devising and optimizing the control of complex systems by for example, perturbing individual dynamics to approach the symmetry point without the need to adjust network structure.

The theoretical paradigm and tools developed here also allow us to address a number of questions, the answers to which could offer further insights into the control of complex networked systems. For example, similar individuals are often accompanied by dense inner connections among them, accounting for the widely observed communities with relatively sparse connections among them in natural and social systems. How such structural property in combination with the similarity and diversity of individual dynamics impacts control is worthy of exploration. Despite the advantage of our tools compared to the other methods in the literature, the network systems that we can address are still the tip of the iceberg, raising the need of new tools based on network science, statistic physics and control theory. At the present, we are incapable of tackling general nonlinear dynamical systems, which is extremely challenging not only in complex networks but also in the canonical control theory. Nevertheless, our approach, we hope, will inspire further interest from physicists and other scientists towards achieving ultimate control of complex networked systems.

## Author Contributions

J.-J.S., Y.-Y.L. and W.-X.W. conceived the research; W.-X.W., Y.-Y.L. and J.-J.S. contributed analytic tools; C.Z. and W.-X.W. performed numerical calculations; W.-X.W. and Y.-Y.L. wrote the paper, J.-J.S. edited the paper.

## Supplementary Material

Supplementary Information Supplementary Information
