# Supplementary material for: Intrinsic dynamics induce global symmetry in network controllability
Source: Sci Rep. 2015 Feb 12;5:8422. doi: 10.1038/srep08422 (PMC4325315; doi:10.1038/srep08422)
Supplement: Supplementary Information [file srep08422-s1.pdf]

Supplementary Materials for

Intrinsic dynamics induce global symmetry in network controllability

Chen Zhao[1], Wen-Xu Wang[1,*], Yang-Yu Liu[2,3,†], Jean-Jacques Slotine[4,5,‡]

[1]School of Systems Science, Beijing Normal University, Beijing, 10085, P. R. China

[2]Channing Division of Network Medicine, Brigham and Women's Hospital,

Harvard Medical School, Boston, Massachusetts 02115, USA

[3]Center for Complex Network Research and Department of Physics,

Northeastern University, Boston, Massachusetts 02115, USA

[4]Nonlinear Systems Laboratory, Massachusetts Institute of Technology,

Cambridge, Massachusetts, 02139, USA

[5]Department of Mechanical Engineering and Department of Brain and Cognitive Sciences,

Massachusetts Institute of Technology,

Cambridge, Massachusetts, 02139, USA

Contents

1 Same controllability for $\Lambda = 0$ and $\Lambda = w_\mathrm{s} I_N$ **2**

2 Efficient approaches for obtaining N_D based on Exact-Controllability Theory **3**

2.1 Dynamic units with 1st-order individual dynamics . . . 3

2.2 The symmetry of N_D . . . 5

2.3 Multiple types of dynamic units with arbitrary order of individual dynamics . . . 7

2.4 Mixture of dynamic units with different orders . . . 7

3 Graphical Approach **8**

3.1 For 1st-order individual dynamics . . . 8

3.2 For high-order individual dynamics . . . 10

3.3 For a mixture of dynamic units with different orders of individual dynamics . . . 11

3.4 Calculation of the cavity method . . . 11

4 Supplementary References **12**

5 Supplementary Figures **13**

1 Same controllability for $\Lambda = 0$ and $\Lambda = w_{\mathbf{s}} I_N$

A linear time-invariant system consisting of N nodes under control can be described by

$$\dot{\mathbf{x}} = A\mathbf{x} + B\mathbf{u}, \tag{S1}$$

where **x** and **u** denote state vector and input vector, respectively, B represents control matrix, describing which nodes are controlled by **u**, A is the state matrix representing the interactions among nodes with $a_{ii} = 0$ $(i = 1, 2, \cdots, N)$ (no self-loops). The controllability matrix of system (S1) is defined as [S1]

$$[B, AB, A^2B, \cdots, A^{N-1}B]. \tag{S2}$$

According to the Kalman's rank condition [S1], if and only if the controllability matrix has full rank, system (S1) is fully controllable.

If we add self-loops with identical weights w_{s} to each node in the network A, system (S1) becomes

$$\dot{\mathbf{x}} = (A + w_{\mathrm{s}} I_N)\mathbf{x} + B\mathbf{u}, \tag{S3}$$

where $\Lambda = w_{\mathrm{s}} I_N$ and I_N is the identity matrix of order N. In order to calculate the controllability matrix of system (S3), we first need to calculate the powers of $A + w_{\mathrm{s}} I_N$, expressed as

$$(A + w_{\mathrm{s}} I_N)^m = A^m + C_m^1 w_{\mathrm{s}} A^{m-1} I_N + C_m^2 w_{\mathrm{s}}^2 A^{m-2} I_N + \cdots + C_m^{m-1} w_{\mathrm{s}}^{m-1} A I_N + w_{\mathrm{s}}^m I_N \tag{S4}$$

with $C_m^i = \frac{m!}{(m-i)!i!}$ and $0! = 1$. We then have

$$(A + w_{\mathrm{s}} I_N)^m B = A^m B + C_m^1 w_{\mathrm{s}} A^{m-1} B + C_m^2 w_{\mathrm{s}}^2 A^{m-2} B + \cdots + C_m^{m-1} w_{\mathrm{s}}^{m-1} AB + w_{\mathrm{s}}^m B. \tag{S5}$$

We can see that $(A + w_{\mathrm{s}} I_N)^m B$ is the linear combination of $B, AB, A^2B, \cdots, A^m B$.

According to Eq. (S5) and the fact that elementary transformation keeps the rank of any matrix invariant, the controllability matrix of system (S3) can be calculated as:

$$\begin{aligned}
& \mathrm{rank}[B, (A + w_{\mathrm{s}} I_N)B, (A + w_{\mathrm{s}} I_N)^2 B, \cdots, (A + w_{\mathrm{s}} I_N)^{N-1} B] \\
= \; & \mathrm{rank}[B, AB + w_{\mathrm{s}} B, A^2 B + 2w_{\mathrm{s}} AB + w_{\mathrm{s}}^2 B, \cdots, A^{N-1} B + (N-1) w_{\mathrm{s}} A^{N-2} B + \cdots \\
& + (N-1) w_{\mathrm{s}}^{N-2} AB + w_{\mathrm{s}}^{N-1} B] \\
= \; & \mathrm{rank}[B, AB, A^2 B + 2w_{\mathrm{s}} AB, \cdots, A^{N-1} B + (N-1) w_{\mathrm{s}} A^{N-2} B + \cdots + (N-1) w_{\mathrm{s}}^{N-2} AB] \\
& \cdots \\
= \; & \mathrm{rank}[B, AB, A^2 B, \cdots, A^{N-1} B],
\end{aligned} \tag{S6}$$

which holds for any w_{s}. Therefore, system (S3) has the same controllability as that of system (S1). In other words, an arbitrary network in the absence of self-loops (presence of self-loops with zero weights) are of the same controllability as that of the network full of self-loops with identical weights.

2 Efficient approaches for obtaining N_D based on Exact-Controllability Theory

We provide details for deriving the unified formulas for N_D of both sparse and dense networks with intrinsic individual dynamics of arbitrary order. Individual dynamics are represented by dynamic units that can be integrated into the network representation, which allows us to use the exact-controllability framework to compute N_D efficiently.

2.1 Dynamic units with 1st-order individual dynamics

In this case, the individual dynamics are characterized by a node with self-loop, the weight of which correspond to the single coefficient in the individual dynamics. First we consider the weight of self-loops can be either zero or w_s. We derive the efficient formulas for sparse and dense networks separately.

(i) Sparse networks with random link weights. Recall that the exact-controllability framework stipulates that the minimum number of driver nodes N_D is determined by the maximum geometric multiplicity $\max_i\{\mu(\lambda_i)\}$ of the eigenvalues λ of Φ:

$$N_\mathrm{D} = \max_i\{\mu(\lambda_i)\} = N - \mathrm{rank}(\lambda^{\max} I_N - \Phi), \tag{S7}$$

where $\lambda^{\max}$ is the eigenvalue corresponding to $\max_i\{\mu(\lambda_i)\}$. Numerical experiments suggest that due to the dominance of diagonal elements to the eigenvalues of sparse matrices with random weights, with high probability $\lambda^{\max}$ will take either 0 or w_s. This results in efficient computation of N_D:

$$N_\mathrm{D} = \begin{cases} N - \mathrm{rank}(\Phi), & \rho_\mathrm{s} < 0.5; \\ N - \min\left\{\mathrm{rank}(\Phi), \mathrm{rank}(\Phi - w_\mathrm{s} I_N)\right\}, & \rho_\mathrm{s} = 0.5; \\ N - \mathrm{rank}(\Phi - w_\mathrm{s} I_N), & \rho_\mathrm{s} > 0.5. \end{cases} \tag{S8}$$

(ii) Dense networks with identical link weight w_l.

We need to first explore the eigenvalues of matrix A. If A is very dense, and all links are of identical weights w_l, it is of high probability to find two of many rows are of the following form:

$$\begin{bmatrix} 0 & w_l & w_l & w_l & \cdots & w_l \\ w_l & 0 & w_l & w_l & \cdots & w_l \\ \cdots & \cdots & \cdots & \cdots & \cdots & \cdots \end{bmatrix}, \tag{S9}$$

where the two nodes corresponding to the two rows are interconnected. The corresponding rows in the

determinant $\det(\lambda I_N - A)$ are

$$\begin{vmatrix} \lambda & -w_l & -w_l & -w_l & \cdots & -w_l \\ -w_l & \lambda & -w_l & -w_l & \cdots & -w_l \\ \cdots & \cdots & \cdots & \cdots & \cdots & \cdots \end{vmatrix}. \tag{S10}$$

We see that if $\lambda = -w_l$, the two rows are identical, indicating that $\det(-w_l I_N - A) = 0$ and $-w_l$ is an eigenvalue of matrix (S9).If a network is very dense, the likelihood to observe two rows with in form shown in matrix (S9) is high, numerical experiments suggest that typically $\lambda^{\max} = -w_l$, resulting in an efficient computation of N_{D}:

$$N_{\mathrm{D}} = N - \mathrm{rank}(A + w_l I_N). \tag{S11}$$

In the presence of nonzero self-loops with weights w_{s}, following similar analysis as shown above, the expected $\lambda^{\max}$ is either $-w_l$ or $w_s - w_l$, yielding an efficient computation of N_{D}:

$$N_{\mathrm{D}} = \begin{cases} N - \mathrm{rank}(\Phi + w_l I_N), & \rho_{\mathrm{s}} < 0.5; \\ N - \min\left\{\mathrm{rank}(\Phi + w_l I_N), \mathrm{rank}[(w_{\mathrm{s}} - w_l) I_N - \Phi]\right\}, & \rho_{\mathrm{s}} = 0.5; \\ N - \mathrm{rank}\big[\Phi + (w_l - w_{\mathrm{s}}) I_N\big], & \rho_{\mathrm{s}} > 0.5. \end{cases} \tag{S12}$$

For more than two types of self-loops, numerical experiments suggest that $\lambda^{\max}$ is the dominating elements in Λ as well. In analogy with the case with two types self-loops, we can still compute N_{D} efficiently.

(I) For sparse networks with random weights,

$$N_{\mathrm{D}} = N - \min_i \left\{\mathrm{rank}\big(\Phi - w_{\mathrm{s}}^{(i)} I_N\big)\right\}. \tag{S13}$$

(II) For dense networks with identical weight w_l,

$$N_{\mathrm{D}} = N - \min_i \left\{\mathrm{rank}\big[\Phi + (w_l - w_{\mathrm{s}}^{(i)}) I_N\big]\right\}. \tag{S14}$$

If one type of self-loop prevails in the network, the comparison is no longer needed and $\lambda^{\max}$ is the weight $w^{\max}$ of the self-loop, reducing the above formulas to

$$N_{\mathrm{D}} = N - \mathrm{rank}\big(\Phi - w_{\mathrm{s}}^{\max} I_N\big), \tag{S15}$$

and

$$N_{\mathrm{D}} = N - \mathrm{rank}\big[\Phi + (w_l - w_{\mathrm{s}}^{\max}) I_N\big], \tag{S16}$$

respectively.

2.2 The symmetry of N_{D}

We consider the symmetry of N_{D} for structured matrix A and in the presence of multiple types of self-loops. Without loss of generality, we denote the matrix

$$\begin{aligned}\Phi &= A+\Lambda \\ &= A+\text{Diag}\left\{\underbrace{w_s^{(1)}\cdots w_s^{(1)}}_{\rho_{\text{s}}^{(1)}},\underbrace{w_s^{(2)}\cdots w_s^{(2)}}_{\rho_{\text{s}}^{(2)}},\cdots,\underbrace{w_s^{(n)}\cdots w_s^{(n)}}_{\rho_{\text{s}}^{(n)}}\right\},\end{aligned} \tag{S17}$$

where $\sum_{t=1}^{n}\rho_{\text{s}}^{(t)}=1$ and we sort $\rho_{\text{s}}^{(t)}$ in descending order $\rho_{\text{s}}^{(1)}\geq\rho_{\text{s}}^{(2)}\geq\cdots\geq\rho_{\text{s}}^{(n)}$. According to Eq. (S13),

$$\begin{aligned}N_{\text{D}} &= N-\text{rank}\left(\Phi-w_{\text{s}}^{(1)}I_N\right) \\ &= N-\text{rank}\left[A+\left(\Lambda-w_{\text{s}}^{(1)}I_N\right)\right].\end{aligned} \tag{S18}$$

Now we exchange two types of self-loops. There are two cases: (i) the exchange does not involve $w_{\text{s}}^{(1)}$ and (ii) $w_{\text{s}}^{(1)}$ is involved.

For case (i), without loss of generality, we consider the exchange between $w_{\text{s}}^{(2)}$ and $w_{\text{s}}^{(n)}$. The matrix $\widetilde{\Phi}$ after the exchange becomes

$$\begin{aligned}\widetilde{\Phi} &= A+\widetilde{\Lambda} \\ &= A+\text{Diag}\left\{\underbrace{w_s^{(1)}\cdots w_s^{(1)}}_{\rho_{\text{s}}^{(1)}},\underbrace{w_s^{(n)}\cdots w_s^{(n)}}_{\rho_{\text{s}}^{(2)}},\cdots,\underbrace{w_s^{(2)}\cdots w_s^{(2)}}_{\rho_{\text{s}}^{(n)}}\right\}.\end{aligned} \tag{S19}$$

According to Eq. (S13),

$$\begin{aligned}\widetilde{N_{\text{D}}} &= N-\text{rank}\left(\widetilde{\Phi}-w_{\text{s}}^{(1)}I_N\right) \\ &= N-\text{rank}\left[A+\left(\widetilde{\Lambda}-w_{\text{s}}^{(1)}I_N\right)\right].\end{aligned} \tag{S20}$$

Since A is a structured matrix and both $\Lambda-w_{\text{s}}^{(1)}I_N$ and $\widetilde{\Lambda}-w_{\text{s}}^{(1)}I_N$ are diagonal matrices with constant entries, both $A+(\Lambda-w_{\text{s}}^{(1)}I_N)$ and $A+(\widetilde{\Lambda}-w_{\text{s}}^{(1)}I_N)$ are typically so-called mixed matrices [S2].

Formally, the concept of mixed matrix is defined as follows. Let $\mathbb{K}$ be a subfield of a field $\mathbb{F}$. For example, $\mathbb{K}=\mathbb{Q}$ (rational numbers) and $\mathbb{F}=\mathbb{R}$ (real numbers). A matrix $M=(M_{ij})$ over $\mathbb{F}$ is called a *mixed matrix* with respect to $(\mathbb{K},\mathbb{F})$ if $M=Q+T$, where Q is a "constant" matrix over $\mathbb{K}$ and T is a "structured" matrix over $\mathbb{F}$, i.e., its entries are either fixed zeros or algebraically independent over $\mathbb{K}$ [S2].

We denote the row set and the column set of M as $\mathcal{R}$ and $\mathcal{C}$, respectively, and the submatrices of Q and T with row set $\mathcal{I}$ and column set $\mathcal{J}$ are written as $Q[\mathcal{I},\mathcal{J}]$ and $T[\mathcal{I},\mathcal{J}]$. A rank identity about mixed matrix M can then be written as:

$$\text{rank}(M) = \max_{\substack{|\mathcal{I}|=|\mathcal{J}| \\ \mathcal{I}\subseteq\mathcal{R},\mathcal{J}\subseteq\mathcal{C}}} \{\text{rank}(Q[\mathcal{I},\mathcal{J}]) + \text{rank}(T[\mathcal{R}\setminus\mathcal{I},\mathcal{C}\setminus\mathcal{J}])\}\,. \tag{S21}$$

In our case, let $Q \equiv \Lambda - w_{\rm s}^{(1)} I_N$ and $\tilde{Q} \equiv \tilde{\Lambda} - w_{\rm s}^{(1)} I_N$. It is easy to show that $\text{rank}(Q[\mathcal{I},\mathcal{J}]) = \text{rank}(\tilde{Q}[\mathcal{I},\mathcal{J}])$ for any $\mathcal{I}\subseteq\mathcal{R},\mathcal{J}\subseteq\mathcal{C}$.

Here, according to the rank identify of mixed matrices [S2], we have

$$\text{rank}\left[A + \left(\Lambda - w_{\rm s}^{(1)} I_N\right)\right] = \text{rank}\left[A + \left(\widetilde{\Lambda} - w_{\rm s}^{(1)} I_N\right)\right], \tag{S22}$$

which indicates $N_{\rm D} = \widetilde{N_{\rm D}}$, assuring the symmetry of $N_{\rm D}$ with respect to exchanging any two types of self-loops except $w_{\rm s}^{(1)}$.

For case (ii), assume that we exchange $w_{\rm s}^{(1)}$ and $w_{\rm s}^{(n)}$. We denote

$$\begin{aligned} \widetilde{\Phi} &= A + \widetilde{\Lambda} \\ &= A + \text{Diag}\left\{ \underbrace{w_s^{(n)} \cdots w_s^{(n)}}_{\rho_{\rm s}^{(1)}}, \underbrace{w_s^{(2)} \cdots w_s^{(2)}}_{\rho_{\rm s}^{(2)}}, \cdots, \underbrace{w_s^{(1)} \cdots w_s^{(1)}}_{\rho_{\rm s}^{(n)}} \right\}. \end{aligned} \tag{S23}$$

According to Eq. (S13),

$$\begin{aligned} \widetilde{N_{\rm D}} &= N - \text{rank}\left(\widetilde{\Phi} - w_{\rm s}^{(n)} I_N\right) \\ &= N - \text{rank}\left[A + \left(\widetilde{\Lambda} - w_{\rm s}^{(n)} I_N\right)\right]. \end{aligned} \tag{S24}$$

It can be simply proved that $\text{rank}(\Lambda - w_{\rm s}^{(1)} I_N) = \text{rank}(\widetilde{\Lambda} - w_{\rm s}^{(n)} I_N)$. Thus according to the rank identify of mixed matrices [S2], we have

$$\text{rank}\left[A + \left(\Lambda - w_{\rm s}^{(1)} I_N\right)\right] = \text{rank}\left[A + \left(\widetilde{\Lambda} - w_{\rm s}^{(n)} I_N\right)\right], \tag{S25}$$

which indicates $N_{\rm D} = \widetilde{N_{\rm D}}$.

The proof of case (i) and case (ii) can be immediately extended to the random distribution of different self-loops in the diagonal. Based on the rank identity of mixed matrices, we can prove the symmetry of $N_{\rm D}$ as well. Taken together, $N_{\rm D}$ is symmetric with respect to exchanging any two types of self-loops in systems with structured matrix A. If A is not a structured matrix, we anticipate that the symmetry of $N_{\rm D}$ still holds with high probability.

2.3 Multiple types of dynamic units with arbitrary order of individual dynamics

The dynamic unit is described by a $d \times d$ matrix

$$\begin{bmatrix} 0 & 1 & 0 & \cdots & 0 & 0 & 0 \\ 0 & 0 & 1 & \cdots & 0 & 0 & 0 \\ 0 & 0 & 0 & \ddots & 0 & 0 & 0 \\ \vdots & \vdots & \vdots & \ddots & \ddots & \vdots & \vdots \\ 0 & 0 & 0 & \cdots & 0 & 1 & 0 \\ 0 & 0 & 0 & \cdots & 0 & 0 & 1 \\ a_0 & a_1 & a_2 & \cdots & a_{d-3} & a_{d-2} & a_{d-1} \end{bmatrix}$$

with $d+1$ non-zero elements, (i.e., $1, a_0, a_1, \cdots, a_{d-1}$) and in general d distinct eigenvalues (except for some pathological cases with zero measure that occur when the values of $a_0, a_1, \cdots, a_{d-1}$ satisfy certain accidental constrains). The whole network system is described by a $dN \times dN$ matrix Φ. The same type of dynamic units results in the same set of eigenvalues, establishing the connection between the number of identical eigenvalues and that of identical units. For this perspective, the eigenvalues of the dynamic units takes over the role of self-loops in the 1st-order individual dynamics. Similar analysis yields efficient computation of N_{D} in terms of the eigenvalues of dynamic units

$$N_{\mathrm{D}} = dN - \min_i \left\{ \mathrm{rank}\big(\Phi - \lambda_d^{(i)} I_{dN}\big) \right\}, \tag{S26}$$

where $\lambda_d^{(i)}$ is any one of the eigenvalues of unit type i with d dimension. Note that different eigenvalues of the same type of dynamic units are typically of the same multiplicity, so any one of them can reflect their impact on N_{D}. The prevalence of a particular dynamic unit leads to a simplified formula

$$N_{\mathrm{D}} = dN - \mathrm{rank}\big(\Phi - \lambda_d^{\max} I_{dN}\big), \tag{S27}$$

where $\lambda_d^{\max}$ is an eigenvalue of the dynamic unit with the largest fraction. The general theory for arbitrary order of individual dynamics is degenerated to that for the special case of 1st-order individual dynamics by setting $d = 1$ and replacing the eigenvalues $\lambda_1^{(i)}$ by the weight $w_{\mathrm{s}}^{(i)}$ of self-loop.

In analogy with the 1st-order dynamics, the symmetry of N_{D} and the highest controllability at the global symmetry point maintain for arbitrary order of individual dynamics and network structure.

2.4 Mixture of dynamic units with different orders

We explore a more general scenario of a mixture of dynamic units of different orders. In this case, the ECT is still applicable insofar as we integrate individual dynamics and network structure into the matrix

form. The efficient theory can be immediately generalized to be used for quantifying n_{D}:

$$N_{\text{D}} = N \sum_d d\rho_d - \min_{d,i} \left\{ \text{rank}\big(\Phi - \eta_d^{(i)} I\big) \right\}, \tag{S28}$$

where $\eta_d^{(i)}$ is the weight of type i self-loop for the 1st-order individual dynamics, and is the eigenvalue of type i dynamic unit for the dth-order individual dynamics, ρ_d is the fraction of dth-order individual dynamics. The prevalence of a particular dynamic unit leads to a simplified formula

$$N_{\text{D}} = N \sum_d d\rho_d - \text{rank}\big(\Phi - \eta^{\max} I\big), \tag{S29}$$

where $\eta^{\max}$ is either the weight of self-loop or the eigenvalue of the prevailing dynamic unit.

3 Graphical Approach

Here, we develop a graphical approach based on maximum matching to quantify the controllability of arbitrary networks with individual dynamics of any order. The cavity method developed in statistical physics has been used to study the maximum matching problem in directed networks [S3], where the unmatched nodes are nothing but the driver nodes.

3.1 For 1st-order individual dynamics

Let's denote the number of matched nodes corresponding to a maximum matching by $N_{\text{m}}(\cdot)$. For a structured matrix A, according to the structural control theory, we have

$$N_{\text{D}}(A) = \max\{1, N - N_{\text{m}}(A)\}. \tag{S30}$$

On the other hand, our general efficient formulas suggest that

$$N_{\text{D}}(A) = \max\{1, N - \text{rank}(A)\}. \tag{S31}$$

Thus we have for structured matrix A

$$\text{rank}(A) = N_{\text{m}}(A). \tag{S32}$$

Since $N_{\text{m}}(A)$ can be analytically solved by the cavity method, rank(A) can then be calculated as well. Due to the fact that matrix rank is required in our efficient theory, Eq. (S32) connects our efficient theory and the cavity method, allowing us to calculate N_{D} by using the tool in statistical physics.

In general, the prerequisite for Eq. (S32) is the dominance of zero in the eigenvalue spectrum of matrix A. In the presence of self-loops of identical (non-zero) weights, according the efficient formula,

$N_{\mathrm{D}} = N - \mathrm{rank}(\Phi - w_{\mathrm{s}} I_N)$, where w_{s} is the weight of the dominant self-loop. Note that the faction of self-loop with w_{s} in matrix Φ is the same as that of zero self-loop in matrix $\Phi - w_{\mathrm{s}} I_N$, ensuring the prevalence of zero eigenvalue in matrix $\Phi - w_{\mathrm{s}} I_N$. Thus the relation between matrix rank and maximum matching still holds, yielding

$$\mathrm{rank}(\Phi - w_{\mathrm{s}} I_N) = N_{\mathrm{m}}(\Phi - w_{\mathrm{s}} I_N), \tag{S33}$$

where $N_{\mathrm{m}}(\Phi - w_{\mathrm{s}} I_N)$ can be numerically calculated by the cavity method as well. Consequently, For multiple types of self-loops integrated with sparse networks, we have

$$N_{\mathrm{D}} = N - \min_i \left\{ N_{\mathrm{m}}\big(\Phi - w_{\mathrm{s}}^{(i)} I_N\big) \right\}. \tag{S34}$$

For the presence of a prevailing self-loop with weight $w_{\mathrm{s}}^{\max}$, Eq. (S34) is reduced to

$$N_{\mathrm{D}} = N - N_{\mathrm{m}}(\Phi - w_{\mathrm{s}}^{\max} I_N) \tag{S35}$$

based on the simplified formula (S15). This analysis is also valid for dense networks with random link weights, but this scenario usually leads to the trivial result of $N_{\mathrm{D}} = 1$, regardless of the configurations of self-loops.

For the special case of dense networks with identical link weights w_l, the prerequisite for employing the cavity method is violated, precluding us from using it directly. This difficulty can be overcome by considering the complement graph of matrix $A + w_l I_N$. We denote the matrix whose elements are all one by J_N and thus the complement graph is given by $J_N - A - w_l I_N$. According to the rank inequality, we have

$$\mathrm{rank}(J_N - A - w_l I_N) \leq \mathrm{rank}(J_N) + \mathrm{rank}(A + w_l I_N), \tag{S36}$$

where the equality holds if one of the ranks in the right hand side is zero. Note that $\mathrm{rank}(J_N) = 1$, which is quite close to zero as compared to N if the network size is large enough. We thus approximately have

$$\mathrm{rank}(J_N - A - w_l I_N) \approx \mathrm{rank}(A + w_l I_N). \tag{S37}$$

The complement graph of the original network is sparse and can be related to the maximum matching as

$$\mathrm{rank}(J_N - A - w_l I_N) \approx N_{\mathrm{m}}(A + w_l I_N), \tag{S38}$$

The sparsity of complement graph enables the use of Eq. (S34) for addressing N_{D} of dense networks with identical link weights and arbitrary types of self-loops, but the weight of self-loops is changed to

$w_l - w_\mathrm{s}$:

$$\begin{aligned} N_\mathrm{D} &= N - \min_i \Big\{ \mathrm{rank}\big[\Phi + (w_l - w_\mathrm{s}^{(i)}) I_N\big] \Big\} \\ &= N - \min_i \Big\{ \mathrm{rank}\big[(A + w_l I_N) + \Lambda - w_\mathrm{s}^{(i)} I_N\big] \Big\} \\ &= N - \min_i \Big\{ N_\mathrm{m}\big[(J_N - A - w_l I_N) + \Lambda - w_\mathrm{s}^{(i)} I_N\big] \Big\} \\ &= N - \min_i \Big\{ N_\mathrm{m}\big[(J_N - A + \Lambda) - (w_l + w_\mathrm{s}^{(i)}) I_N\big] \Big\}, \end{aligned} \tag{S39}$$

where A is dense, and $J_N - A$ except diagonal becomes sparse, ensuring the applicability of the cavity method. In the presence of a prevailing self-loop with weight $w_\mathrm{s}^{\max}$, Eq. (S39) is reduced to

$$N_\mathrm{D} = N - N_\mathrm{m}\big[(J_N - A + \Lambda) - (w_l + w_\mathrm{s}^{\max}) I_N\big]. \tag{S40}$$

3.2 For high-order individual dynamics

For high-order individual dynamics, the eigenvalues of dynamic units determine N_D. Due to the regular configurations of dynamic units, the relationship between the maximum matching and the rank of the $dN \times dN$ network matrix is violated, so that the cavity method for the 1st-order individual dynamics cannot be extended to the high-order cases directly. The key to implementing the cavity method lies in offering a general scheme to construct an equivalent network with 1st-order individual dynamics that shows the same N_D as original system. Note that since the state matrix with high-order individual dynamics must be sparse, the role of links can be regarded as the perturbation to the eigenvalues of dynamic units. We map each dynamic unit to a single node with self-loop, the weight of which is set to be an eigenvalue of the unit's state matrix. The interactions among units in the original network can be preserved by linking the correspondent single nodes. The network construction reflects the role of dynamic units in N_D and the perturbation of interactions among them in a network consisting of N nodes with self-loops, offering a general mapping of networks with high-order individual dynamics into 1st-order network systems so as to use the cavity method (S34). To be concrete, for a network system with dth-order individual dynamics, N_D can be calculated via

$$N_\mathrm{D} = N - \min_i \Big\{ N_\mathrm{m}\big(\Phi'_N - \lambda_d^{(i)} I_N\big) \Big\}, \tag{S41}$$

where $N_\mathrm{m}(\cdot)$ is the maximum matching, Φ'_N is the reduced state matrix with N nodes and $\lambda_d^{(i)}$ is the one of the eigenvalues of type-i dynamic unit's state matrix. In the presence of a prevailing dynamic unit of dth order, we denote one of the eigenvalues of the dynamic unit as $\lambda_d^{\max}$, Eq. (S41) is reduced to

$$N_\mathrm{D} = N - N_\mathrm{m}\big(\Phi'_N - \lambda_d^{\max} I_N\big). \tag{S42}$$

3.3 For a mixture of dynamic units with different orders of individual dynamics

Based on the efficient theory (S28) for a mixture of individual dynamics, we have

$$N_{\rm D} = N - \min_{d,i}\left\{N_{\rm m}\big(\Phi'_N - \eta_d^{(i)} I_N\big)\right\}, \tag{S43}$$

where Φ'_N is the reduced state matrix with N nodes and $\eta_d^{(i)}$ is either the weight of self-loops for the 1st-order individual dynamics or one of the eigenvalues of dth-order individual dynamics. If there exists a dynamic unit of dth order prevails in the network, Eq. (S43) is reduced to

$$N_{\rm D} = N - N_{\rm m}\big(\Phi'_N - \eta_d^{\max} I_N\big), \tag{S44}$$

where $\eta_d^{\max}$ is either the self-loop of the dynamic unit if $d = 1$, or one of the eigenvalues of the dynamic unit's state matrix.

3.4 Calculation of the cavity method

In Supplemental Materials of Ref. [S3], the cavity method for maximum matching of directed networks is detailed. Specifically, for a directed network with in- and out-degree distributions $\hat{P}(k_{\rm in})$ and $P(k_{\rm out})$, respectively, the density of driver nodes is given by

$$n_{\rm D} = \frac{1}{2}\left\{\big[G(\widehat{w}_2) + G(1-\widehat{w}_1) - 1\big] + \big[\widehat{G}(w_2) + \widehat{G}(1-w_1) - 1\big] + \frac{z}{2}\big[\widehat{w}_1(1-w_2) + w_1(1-\widehat{w}_2)\big]\right\}, \tag{S45}$$

where $z = \langle k \rangle$ is the mean degree and the generating functions $G(x)$ and $\widehat{G}(x)$ are given by

$$G(x) = \sum_{k_{\rm out}=0}^{\infty} P(k_{\rm out}) x^{k_{\rm out}}, \tag{S46}$$

$$\widehat{G}(x) = \sum_{k_{\rm in}=0}^{\infty} \widehat{P}(k_{\rm in}) x^{k_{\rm in}}. \tag{S47}$$

The quantities w_1, w_2, $\widehat{w}_1$ and $\widehat{w}_2$ in Eq. (S45) can be solved by the following set of self-consistent equations:

$$w_1 = H(\widehat{w}_2), \tag{S48}$$

$$w_2 = 1 - H(1-\widehat{w}_1), \tag{S49}$$

$$\widehat{w}_1 = \widehat{H}(w_2), \tag{S50}$$

$$\widehat{w}_2 = 1 - \widehat{H}(1-w_1), \tag{S51}$$

where the generating functions are defined as

$$H(x) = \sum_{k_{\rm out}=0}^{\infty} Q(k_{\rm out}+1) x^{k_{\rm out}}, \tag{S52}$$

$$\widehat{H}(x) = \sum_{k_{\rm in}=0}^{\infty} \widehat{Q}(k_{\rm in}+1) x^{k_{\rm in}}, \tag{S53}$$

where the terms

$$Q(k_{\text{out}}) \equiv \frac{k_{\text{out}} P(k_{\text{out}})}{\langle k_{\text{out}} \rangle}, \tag{S54}$$

$$\widehat{Q}(k_{\text{in}}) \equiv \frac{k_{\text{in}} \widehat{P}(k_{\text{in}})}{\langle k_{\text{in}} \rangle}. \tag{S55}$$

The maximum matching in Eqs. (S34), (S35), (S39), (S40), (S41), (S42), (S43) and (S44) can be analytically calculated by the cavity method in terms of solving the the set of self-consistent equations. For example, the maximum matching $N_{\text{m}}(\Phi - w_{\text{s}}^{(i)} I_N)$ in Eq. (S34) can be determined by calculating $n_{\text{D}}(\cdot)$ of matrix $\Phi - w_{\text{s}}^{(i)} I_N$ based on the cavity method in the sense that

$$N_{\text{m}}(\Phi - w_{\text{s}}^{(i)} I_N) = N - N \cdot n_{\text{D}}(\Phi - w_{\text{s}}^{(i)} I_N). \tag{S56}$$

To acquire $n_{\text{D}}(\Phi - w_{\text{s}}^{(i)} I_N)$, we need the in- and out-degrees k_{in} and k_{out} of nodes. In this case, k_{in} and k_{out} are calculated from the matrix $\Phi - w_{\text{s}}^{(i)} I_N$ rather than from the original state matrix. Thus we should first define self-loops for this matrix. In particular, if a diagonal element is nonzero, the relevant node corresponding to the nonzero element contains a self-loop. As a result, the in- and out-degree of the node in the original network A is increased by 1; for other nodes in the absence of self-loops, their in- and out-degrees are the same as those in the original network. We thus obtain a set of new k_{in} and k_{out}. Using the information of node degrees, we can solve the set of self-consistent equations, which together with the generating functions gives n_{D} and consequently N_{m}. Finally, Eqs. (S34), (S35), (S39), (S40), (S41), (S42), (S43) and (S44) based on the maximum matching can be calculated based on the graphical approach. The results obtained from the graphical approach have been validated in Supplementary Fig. S1, S2 and S3, as compared to those obtained from the efficient formulas.

4 Supplementary References

[S1] Kalman, R. E. Mathematical description of linear dynamical systems. *J. Soc. Indus. Appl. Math. Ser. A* **1**, 152-192 (1963).

[S2] Murota, K. *Matrices and Matroids for Systems Analysis* (Springer Heidelberg Dordrecht London, New York, USA, 2010).

[S3] Liu, Y.-Y., Slotine, J.-J. & Barabási, A.-L. Controllability of complex networks. *Nature* **473**, 167-173 (2011).

5 Supplementary Figures

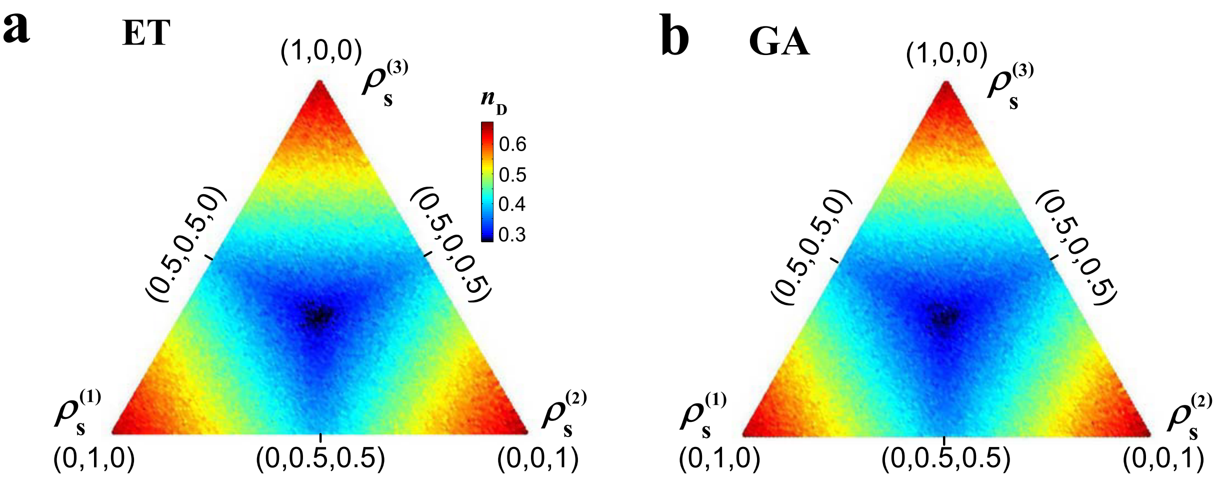

Supplementary Figure S1: The symmetry of controllability in the presence of three types of self-loops. n_D with three types of randomly distributed self-loops s_1, s_2 and s_3 in ER networks obtained by the efficient tool (ET) (**a**) and the graphical approach (GA) (**b**). The color bar denotes the value of n_D and the coordinates in the triangle stands for $\rho_\mathrm{s}^{(1)}$ $\rho_\mathrm{s}^{(2)}$ and $\rho_\mathrm{s}^{(3)}$. The results obtained from GA in (**a**) and (**b**) are based on Eq. (S13). The results obtained from CM is based on Eq. (S34). The network size N is 2000. All the results are obtain over 100 independent realizations.

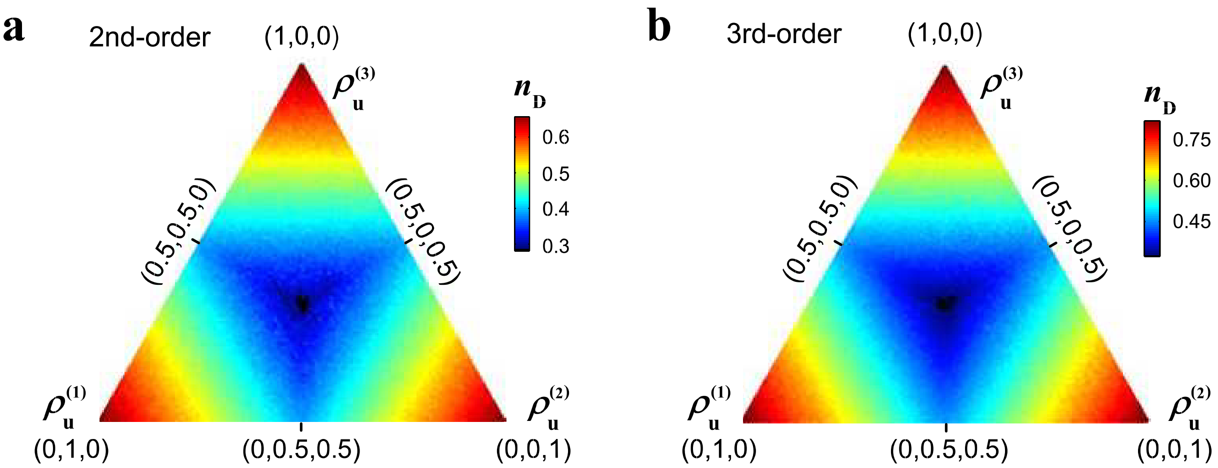

Supplementary Figure S2: Controllability associated with three types of high-order dynamic units obtained by the graphical approach. a-b, n_{D} as a function of the density $\rho_{\mathrm{u}}^{(1)}$, $\rho_{\mathrm{u}}^{(2)}$ and $\rho_{\mathrm{u}}^{(3)}$ of three types of dynamic units belonging to the 2nd-order individual dynamics (**a**) and the 3rd-order individual dynamics (**b**). The results obtained from graphical approach are based on Eq. (S41). The directed ER network size N for the 2nd-order individual dynamics is 1000 and for the 3rd-order ones is 500. All the results are obtained from 50 independent realizations.

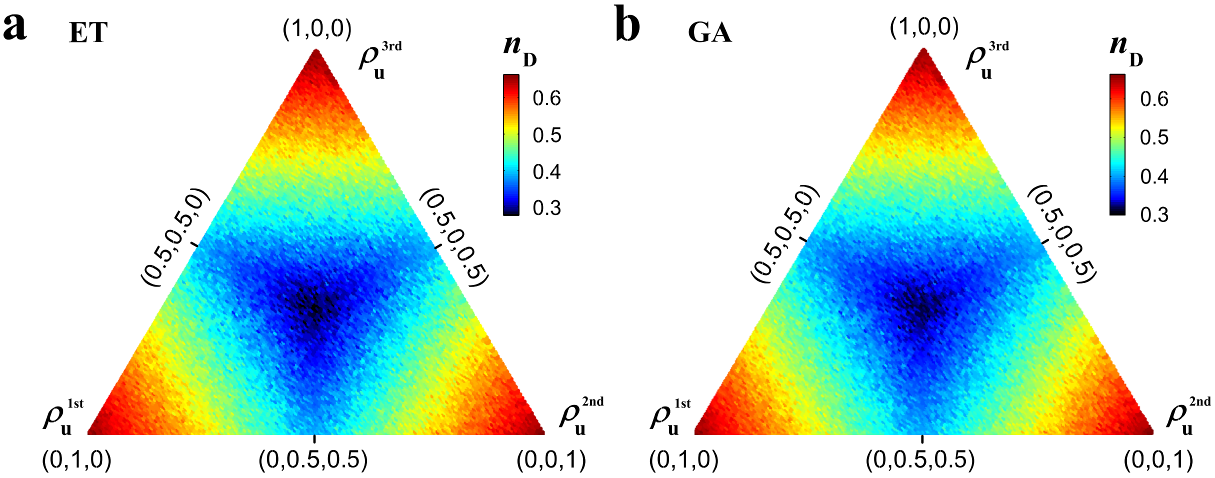

Supplementary Figure S3: Controllability for a mixture of dynamic units with different orders. a-b, n_D as a function of the ρ_u^{1st}, ρ_u^{2nd} and ρ_u^{3rd} of the 1st-, 2nd- and 3rd-order individual dynamics obtained by using the ET (**a**) and CM (**b**). Here the sum of the fractions of dynamic units with different orders is normalized. ET denotes the results from the general formulas (S26), and CM denotes the results from the graphical approach corresponding to Eq. (S41). The directed ER network size N in **a** is 1000 and in **b**-**d** is 500. All the results are obtained from 50 independent realizations.
